# Epicardial fat volume evaluated with multidetector computed tomography and other risk factors for prevalence of three-vessel coronary lesions

**DOI:** 10.1186/s40001-022-00956-w

**Published:** 2022-12-27

**Authors:** Bulang Gao, Caiying Li, Qibin Liao, Tong Pan, Chunfeng Ren, Qinying Cao

**Affiliations:** 1 Department of Neurosurgery, Shijiazhuang People’s Hospital, 365 South Jianhua Street, Shijiazhuang, 050011 Hebei China; 2 Department of Medical Imaging the Second Hospital, Hebei Medical University, Shijiazhuang, 050011 Hebei China; 3 Department of Laboratory Analysis, The First Affiliated Hospital of Zhengzhou University, 1 Longhu Middle Ring Road, Zhengzhou, 450018 Henan China

**Keywords:** Three-vessel coronary lesion, Computed tomography, Epicardial fat, Risk factor, Biochemical marker

## Abstract

**Purpose:**

To retrospectively investigate the epicardial fat volume with multidetector computed tomography (MDCT) and other risk factors for the prevalence of three-vessel coronary lesion.

**Materials and methods:**

MDCT was performed on 424 subjects with or without three-vessel coronary lesion. Blood was tested for triglyceride, high-density lipoprotein (HDL), low-density lipoprotein (LDL), apolipoprotein A (ApoA), apolipoprotein B (ApoB), alanine aminotransferase (ALT), aspartate aminotransferase (AST), lipoprotein a, and fasting blood glucose.

**Results:**

Among all the subjects, a significant (*P* < 0.05) negative linear correlation existed between age and ALT or ALT/AST. The epicardial fat had a significant (*P* < 0.05) negative linear correlation with HDL and Apo A but a positive correlation with age and ApoB/ApoA. The epicardial fat volume and the fasting blood glucose were significantly (*P* = 0.001) greater in the patients than in the control group, whereas HDL and Apo A were both significantly (*P* < 0.0001) smaller in the patients than in the control groups. A significant prediction value (*P* < 0.05) existed in age increase, male gender, epicardial fat increase, low HDL, high LDL, and elevated fasting blood glucose.

**Conclusion:**

Three-vessel coronary lesions are more prevalent in subjects with greater volume of epicardial fat and in male gender.

## Introduction

Cardiovascular disease is increasing day by day because of changes in lifestyle and lack of exercise (sedentary habits) and increasingly threatens human health as one of the major diseases [1–3]. Myocardial infarction is an important cause of premature death, and a three-vessel coronary lesion is extremely dangerous for acute myocardial infarction. The three-vessel coronary lesion is defined as severe stenosis (over 75%) presented in three major coronary arteries of the right or left coronary artery trunk, left anterior descending, or left circumflex branches. It is crucial to early diagnose three-vessel coronary lesion for the prevention of possible myocardial infarction. Epicardial fat is the true visceral adipose tissue that covers the cardiac surface and coronary arteries within the pericardium [4, 5]. Although little is known regarding the pathophysiologic and metabolic properties and role of epicardial fat, it has been indicated in the initiation and development of coronary atherosclerosis [6–8]. The epicardial fat is thicker in patients with coronary artery diseases than in those with normal coronary arteries [9] and can predict cardiovascular events in subjects with atypical coronary artery disease [10, 11]. Evidence is accumulating that the epicardial fat may act as an endocrine organ because of their comparable patterns of adipocytokine production and may be associated with the development of coronary atherosclerosis through several paracrine mechanisms including local inflammatory mediators which trigger the atherosclerotic process, and other systemic effects [12–14]. The risk factors for myocardial infarction have been well studied and some risk factors have been identified including hypertension, dyslipidemia, smoking, and diabetes. However, the risk factors for three-vessel coronary lesions have not been studied especially with regard to the epicardial fat. This study investigated the relationship of three-vessel coronary lesions with the epicardial fat volume evaluated with multidetector computed tomography (MDCT) and other biochemical factors, trying to establish some risk factors for predicting three-vessel coronary lesion.

## Materials and methods

### Subjects

This retrospective cross-sectional one-center study was approved by the ethics committee of our hospital, and all patients had signed the informed consent to participate. All methods were performed in accordance with the relevant guidelines and regulations. Patients who had cardiac CT angiography between July and December 2019 in our hospital were recruited in this study. The inclusion criteria were over 75% stenosis in three coronary arteries of a left anterior descending branch, left circumflex branch, and the trunk of the right or left coronary artery, with no valvular heart disease, no metabolic or blood diseases, or no history of infectious or inherited diseases. Two hundred and six patients met the inclusion criteria and were enrolled as the patient group including 106 males and 100 females with an age range of 39–83 years (mean 54.4 ± 10.3). Two hundred and eighteen healthy people matched in age and sex were chosen as the control group who had no stenosis or plaques in the coronary arteries including 110 males and 108 females with an age range of 25–77 years (mean 52.7 ± 9.4).

### MDCT angiography and quantitative evaluation

**Fig. 1:**
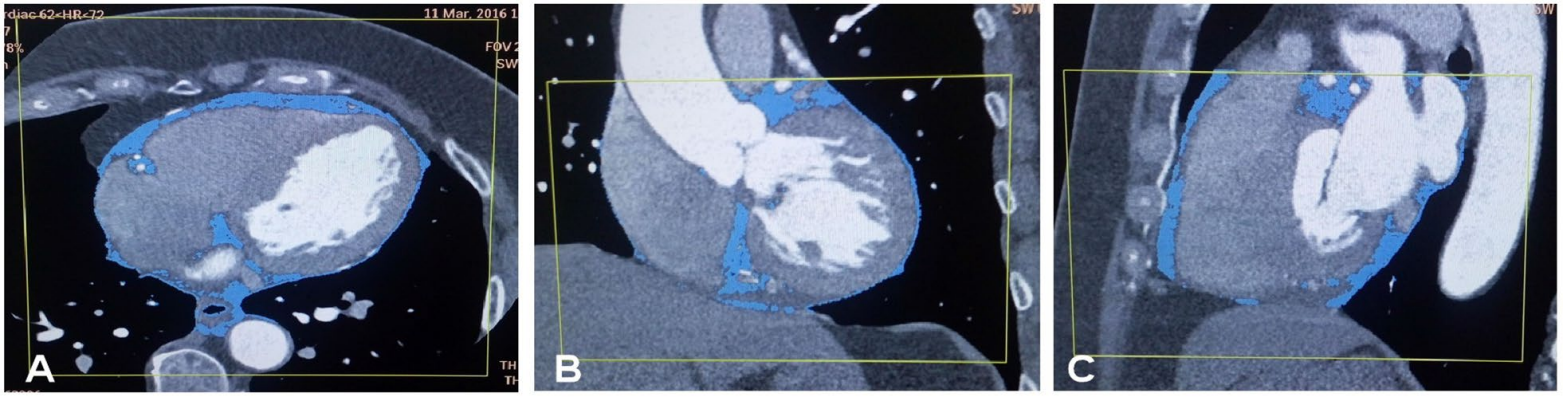
Measurement of the epicardial fat volume in **A** (cross section) and **B** (sagittal position). The epicardial fat indicates the fat which has the Hounsfield units between − 33– − 190 Hu within the pericardium

A 256-slice CT scanner (Brilliance iCT, Philips Healthcare, Cleveland, OH, USA) was used for cardiac MDCT angiography using the following parameters: detector collimation 128 × 0.625 mm, tube current 250–350 mAs, tube voltage 80–120 kV, pitch 0.18, matrix 512 × 512, gantry rotation time 330 ms, and field of view 250 mm. The angiography was performed with the ECG-gated technique during a breath hold of 4–7 s. The contrast medium iohexol (0.8 ml/kg) was injected intravenously at a rate of 4–5 ml/s with a double-tube high-pressure syringe. The MDCTA scanning field was from 0.5 cm below the tracheal bifurcation to the superior border of the liver. The scanning raw data were reconstructed with 75% of RR wave for the right and left coronary arteries and their primary branches and transferred to the Philips EBW 4.5 workstation (Extended BrillianceTM Workspace, V4.5.2.4031, Philips Healthcare Nederland B.V., The Netherlands) for further analysis using specialized software (Vitrea 2; Vital Images, Inc., Minneapolis, MN, USA). Techniques including multiplanar reconstruction, volume rendering, surface reconstruction and maximum intensity projection were applied for the assessment of stenosis in the coronary trunk and branches and quantitative measurement of pericardial fat. Severe stenosis of the coronary was diagnosed as over 75% stenosis. The epicardial fat was measured by depicting the outer margin of the pericardium between the inferior border of the pulmonary artery and the upper border of the diaphragm, and fat within the pericardium was defined as the epicardial fat which was assessed three-dimensionally in the volume according to the fat Hounsfield units between − 30 and − 190 Hu (Fig. 1). Abnormal increase of epicardial fat was defined as the epicardial fat volume greater than 90.0 ml. The body mass index (BMI) for Chinese people was calculated as BMI = body weight (kg)/height (m) [2].

### Laboratory test

Blood was drawn after 8 h overnight fasting for testing of triglyceride (TG), high-density lipoprotein (HDL), low-density lipoprotein (LDL), apolipoprotein A (Apo A), apolipoprotein B (Apo B), Alanine aminotransferase (ALT), Aspartate aminotransferase (AST), lipoprotein a (Lpa), and fasting blood glucose. For the Chinese population, the following values were considered abnormal: TG > 1.70 mmol/L, HDL < 1.04 mmol/L, LDL > 3.37 mmol/L, Apo A < 1.08 g/L, Apo B > 1.17 g/L, Lpa > 30 mg/dl, fasting blood glucose  > 6.10 mmol/L, ALT > 50.00 U/L, and AST > 40.00 U/L. Age increase was defined from young (younger than 45 years) and middle-aged (45–60 years) to elderly people (> 60 years) to detect the effect of age increase on the prevalence of stenosis in three coronary arteries.

### Statistical analysis

The JMP 10.0 statistical software (SASS, Chicago, IL, USA) was used for statistical analysis. All continuous data were expressed as mean ± standard deviation (SD). Student’s *t* test was used for comparison between the patient and healthy groups, and a linear logistical analysis was performed for correlation analysis. *P* < 0.05 was set as the statistical significance.

## Results

**Fig. 2:**
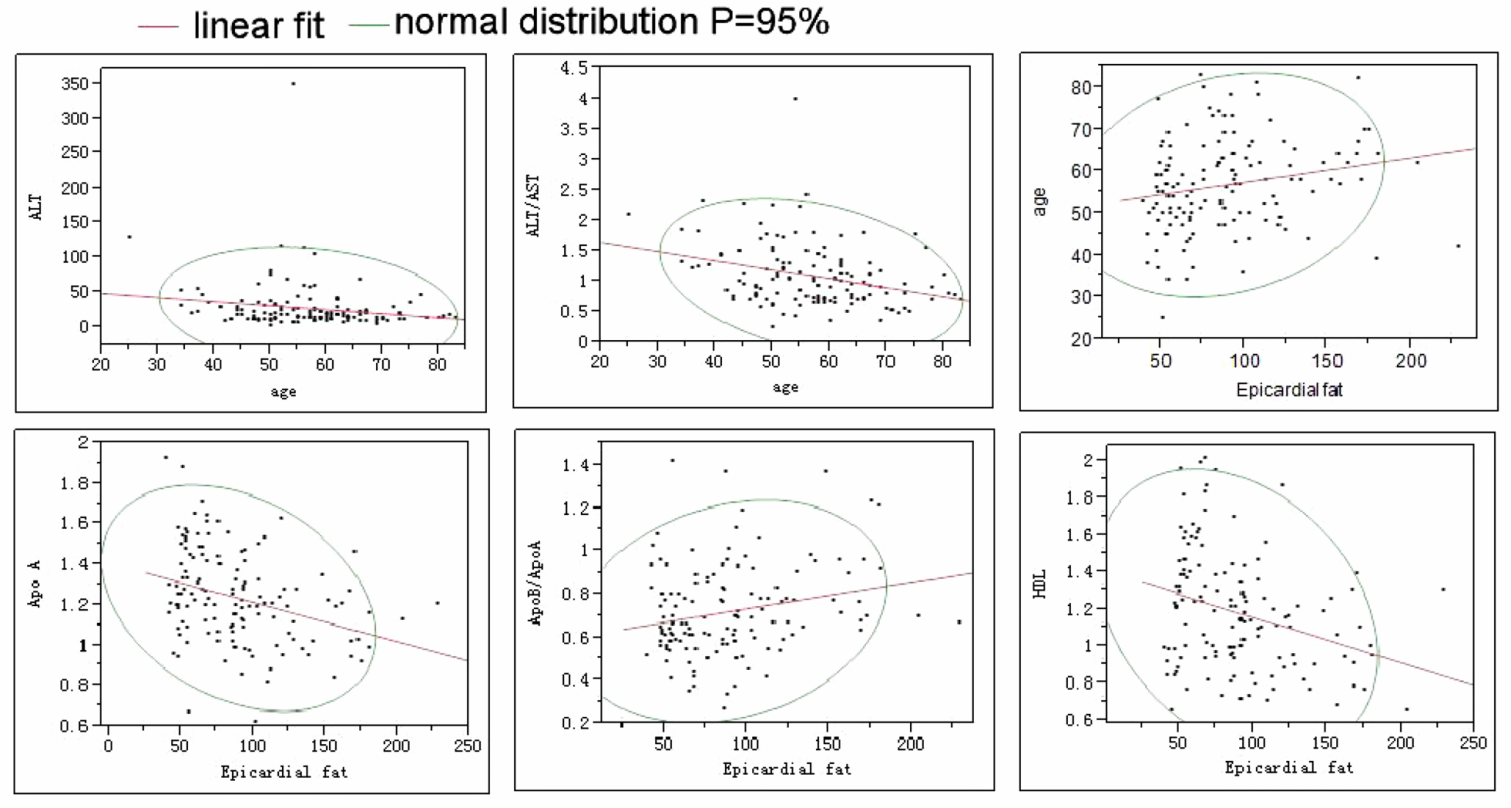
A significant (*P* < 0.05) negative correlation existed between age and alanine aminotransferase (ALT) or ALT/AST (aspartate aminotransferase). The epicardial fat has a significant (*P* < 0.05) negative correlation with either apolipoprotein A (Apo A) or high-density lipoprotein (HDL) but a significant (*P* < 0.05) positive correlation with either age or Apo B/Apo A. The central line indicates linear fit and the circle indicates the bivariate normal ellipse (*P* = 0.95)

**Fig. 3:**
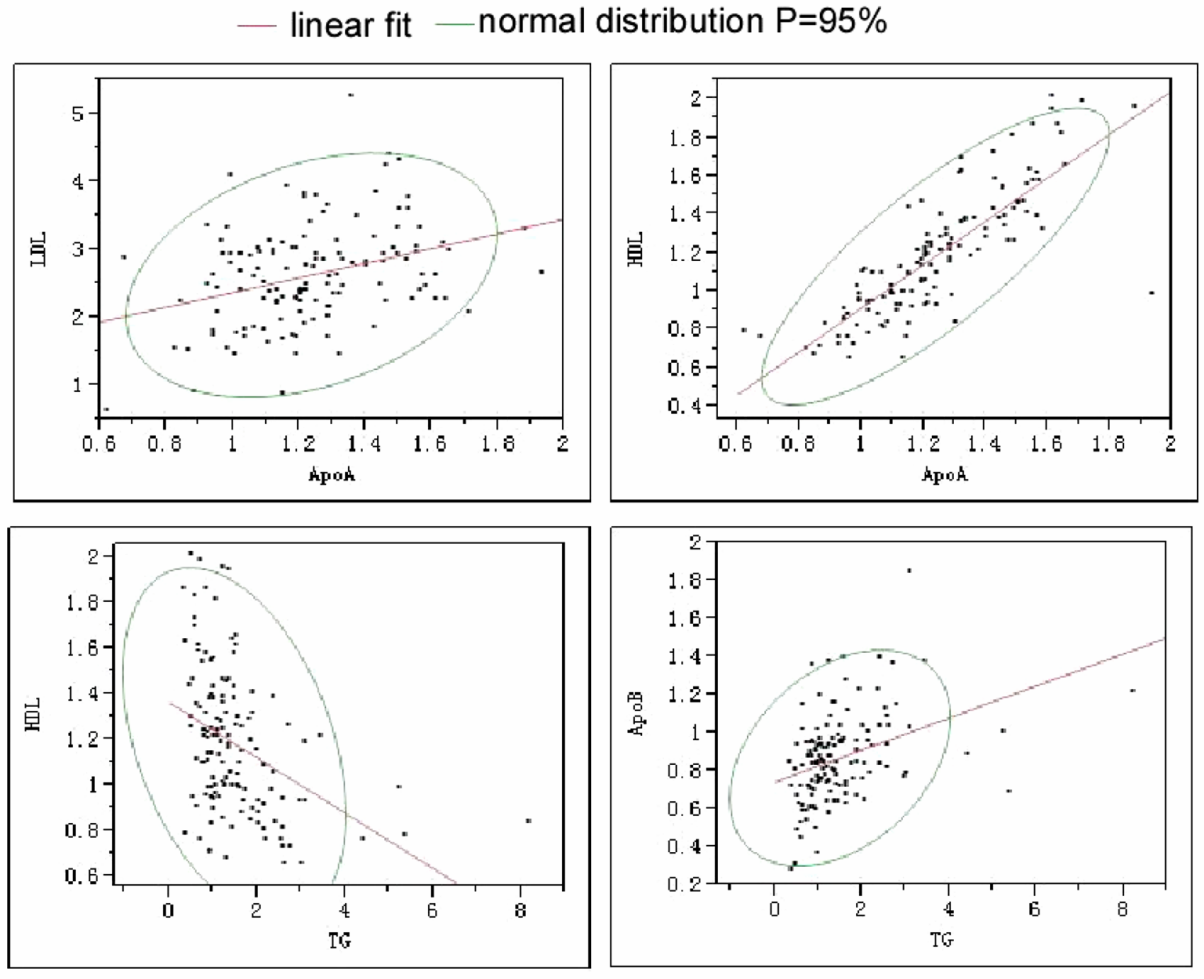
A significant (*P* < 0.05) positive correlation existed between high-density lipoprotein (HDL) and low-density lipoprotein (LDL). Triglyceride (TG) has a significant (*P* < 0.05) negative correlation with HDL but a positive correlation with apolipoprotein B (Apo B). The central line indicates linear fit and the circle indicates the bivariate normal ellipse (*P* = 0.95)

Among all the subjects, a negative linear correlation existed between age and ALT (R = 0.18, *P* = 0.039) or ALT/AST (R = 0.31, *P* = 0.0003). The epicardial fat had a negative linear correlation with HDL (R = 0.31, *P* = 0.0003) or Apo A (R = 0.33, *P* < 0.0001) but a positive correlation with age (R = 0.21, *P* = 0.017) and Apo B/Apo A (R = 0.23, *P* = 0.008) (Fig. 2). A positive linear correlation existed between Apo A and Apo B (R = 0.23, P = 0.009), HDL (R = 0.82, *P* < 0.0001), LDL (R = 0.34, *P* < 0.0001) or fasting blood glucose (R = 0.25, *P* = 0.004) (Fig. 3). TG had a positive linear correlation with Apo B (R = 0.38, P < 0.0001) but a negative linear correlation with HDL (R = 0.40, *P* < 0.0001) (Fig. 3). AST had a positive linear correlation with ALT (R = 0.76, *P* < 0.0001).

**Fig. 4:**
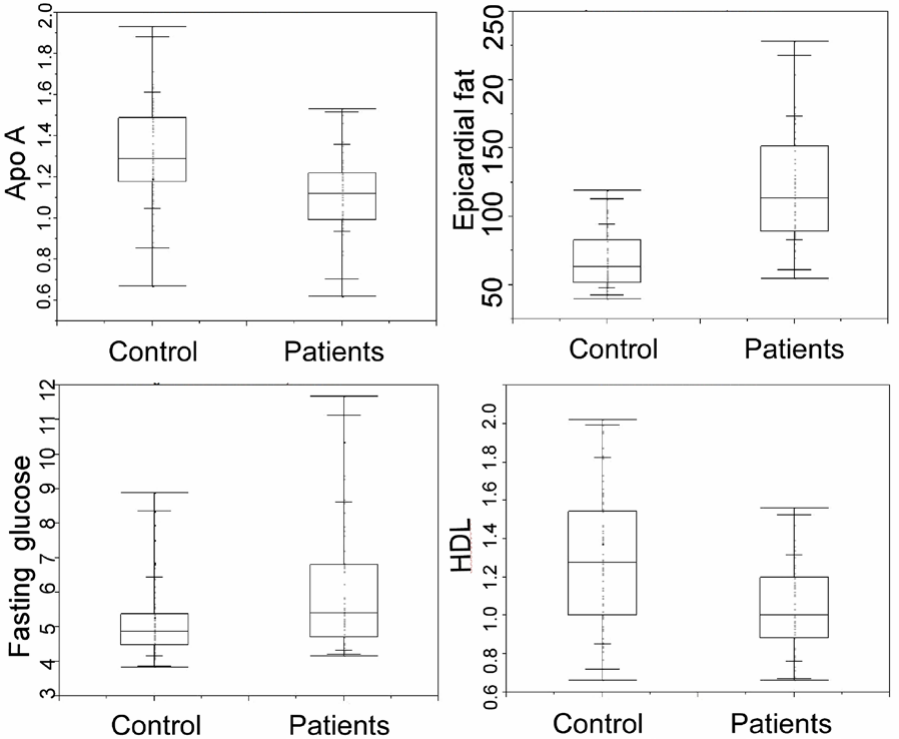
A significant (*P* < 0.05) difference was detected in the epicardial fat volume, high-density lipoprotein (HDL), apolipoprotein A (Apo A), and fasting blood glucose between the control and the patient group

Between the patient and healthy groups, a significant (*P* < 0.05) difference was detected in the epicardial fat volume, HDL, Apo A, and fasting blood glucose (Fig. 4). The epicardial fat volume and the fasting blood glucose were significantly (*P* = 0.001) greater in the patient than in the healthy group, whereas the HDL and Apo A were both significantly (*P* < 0.0001) smaller in the patient than in the healthy groups (Table 1).

**Table 1:** Data comparison between the patient and healthy groups (mean ± SD, n = 134)

	Healthy (n = 78)	Patient (n = 56)	*P*
Age	52.744 ± 9.423	54.446 ± 10.322	0.65
Epicardial fat	66.941 ± 19.156	120.654 ± 37.615	0.000
TG	1.399 ± 1.057	1.596 ± 1.003	0.277
HDL	1.294 ± 0.334	1.035 ± 0.213	0.000
LDL	2.690 ± 0.633	2.563 ± 0.862	0.352
Apo A	1.316 ± 0.230	1.123 ± 0.175	0.000
Apo B	0.854 ± 0.196	0.893 ± 0.277	0.369
ALT	29.429 ± 43.605	25.695 ± 20.793	0.557
AST	22.083 ± 14.887	23.316 ± 15.396	0.644
Lpa	27.692 ± 30.649	29.670 ± 29.737	0.710
FBG	5.135 ± 0.993	6.040 ± 1.735	0.001

SD, standard deviation; TG, triglyceride; HDL, high density lipoprotein; LDL, low density lipoprotein; Apo A, apolipoprotein A; Apo B, apolipoprotein; ALT, alanine aminotransferase; AST, aspartate aminotransferase; Lpa, lipoprotein a; FBG, fasting blood glucose

After analysis of the relationship of stenosis prevalence in three coronary arteries as the dependent variable with the independent variables of age increase, male gender, epicardial fat increase, high LG, high LDL, low HDL, low Apo A, High Apo B, increased ALT and AST, high Lpa, and increased fasting blood glucose, a significant prediction value (*P* < 0.05) existed in age increase (odds ratio (OR) = 6.34, *P* < 0.0001), male gender (OR = 8.36, *P* = 0.001), epicardial fat increase (OR = 14.83, *P* < 0.001), low HDL (OR = 4.74, *P* = 0.01), high LDL (OR = 4.89, *P* = 0.05), and elevated fasting blood glucose (OR = 4.69, *P* = 0.04). For male patients, age increase (OR = 7.1, P = 0.001), epicardial fat increase (OR = 11.4, *P* = 0.004), low HDL (OR = 4.8, P = 0.50) and elevated fasting blood glucose (OR = 17.2, *P* = 0.03) all had a significant prediction value (*P* < 0.05) for the prevalence of stenosis in three coronary arteries. However, for female patients, only age increase (OR = 4.5, *P* = 0.03) and epicardial fat (OR = 21.8, *P* < 0.0001) had significant prediction value for the prevalence of stenosis in three arteries.

## Discussion

In this study, we investigated the relationship of three-vessel coronary lesions with epicardial fat and other biochemical factors and identified some risk factors for three-vessel coronary lesions including age increase, male gender, epicardial fat increase, low HDL, high LDL, and increased fasting blood glucose. Some factors like Apo A and B and TG exerted an indirect effect on the development of three-vessel coronary lesion through direct risk factors of HDL, LDL, and fasting blood glucose.

In investigating the relationship of pericardial fat with angiographic coronary artery stenosis, some inconsistency was encountered. Some studies demonstrated a positive correlation between the thickness of the epicardial adipose tissue measured by echocardiography and the severity of coronary artery stenosis [15–17]. On the contrary, another study showed no association between epicardial fat thickness evaluated with echocardiography and coronary artery stenosis [18]. The difference may be caused by different measurement techniques (echocardiography and MDCT) and study cohorts. In comparison to echocardiography, MDCT has high temporal and spatial resolution, three-dimensional views, and submillimeter collimation, and is more sensitive and specific in measuring the fat thickness in deeper epicardial fat and the thickest part in the atrioventricular grooves [19]. In our study, we used 256-slice MDCT in measuring the epicardial fat volume and found its association with a three-vessel coronary lesion. The three-vessel coronary lesion and its relationship with epicardial fat and other risk factors have not been fully investigated.

It has been reported that epicardial fat and visceral adipose tissue can secret proinflammatory and proatherogenic adipocytokines including TNF-α, interleukin-1,6, and monocyte chemo-attractant protein-1 [13, 14, 20, 21]. However, adiponectin is reduced with an increase of fat, and adiponectin has an anti-inflammatory effect via inhibition of NF-κB activity and TNF-α [22, 23] and is inversely associated with mixed and noncalcified plaque formation [24]. Moreover, epicardial adipose tissue can secret greater amounts of inflammatory cytokines and has more inflammatory cell infiltration than the subcutaneous fat in the legs [14, 20, 21]. Our finding of a positive correlation between the epicardial fat and three-vessel coronary lesion supports the theory that epicardial fat has a devastating effect on coronary atherosclerosis via the inflammatory processes.

The initiation of atherosclerosis is the deposition and retention of atherogenic lipoprotein particles in the wall of a susceptible coronary artery, which is followed by reactive inflammation, smooth muscle cell proliferation, fibrosis, and calcification [25, 26]. In contrast, those lipoproteins associated with reverse cholesterol transport are able to clean out excess cholesterol from macrophages in atherosclerotic lesions, thus offering an atheroprotective effect. The lipoprotein particles consist of lipid components, including cholesterol, cholesterylester, phospholipids, and triglycerides, and protein components like Apo A, B, C, and E. The critical mechanism of the atherogenic dyslipidemia paradigm is that the lipoprotein particles contained by Apo B are atherogenic due to the physical binding of Apo B to proteoglycans in the arterial wall while the HDL particles contained in Apo A are atheroprotective through removing cholesterol from macrophages in the arterial wall and preventing LDL oxidation and maladaptive inflammation [27]. Apo A is mainly synthesized by the liver and intestines and secreted into blood circulation. In the circulation, Apo A undergoes some remodeling processes facilitated by some enzymes including plasma lipid transfer protein, lecithin-cholesterol acyltransferase, and cholesterol ester transfer protein and finally matures to more lipid-rich and larger HDL particles before performing its atheroprotective function [27]. This theory explains our findings that the epicardial fat volume is negatively correlated with HDL or Apo A but positively correlated with Apo B/Apo A. TG is positively correlated with Apo B but negatively correlated with HDL, and thus, TG may indirectly affect the epicardial fat and consequently the prevalence of three-vessel coronary lesions through affecting Apo B and HDL. AST and ALT reflect the function of the liver, and with hepatic function changes, AST and ALT together with HDL and Apo A may alter in content and function. Finally, the epicardial fat is affected to influence the coronary lesion. The vascular endothelium has an important regulatory role in maintaining homeostasis by providing a physical barrier between the vessel wall and its luminal contents and secreting mediators regulate vascular tone. The endothelial cells also interact with circulating proteins and cells to adjust platelet adhesion, coagulation and fibrinolysis, and adherence of leucocytes to the endothelial cell surface [28]. Hyperglycemia may result in endothelial dysfunction, promoting vasospasm, thrombosis, and inflammation, which are implicated in the early stages of atherosclerotic disease. Moreover, hyperglycemia may be accompanied by increased LDL triglyceride and decreased HDL to influence epicardial fat and coronary artery lesions.

Our study showed that males have more risk factors for the development of three-vessel coronary lesions than females and that the male gender is a risk factor for this development. Studies have shown that cardiovascular diseases are more prevalent in men and that men have a greater incidence of coronary heart disease [3, 29]. Generally speaking, cardiovascular disease presentation in females is delayed by approximately 10 years compared with males [30]. Sex hormones may exert metabolic and hemodynamic effects and result in the gender difference in the relevance of risk factors in determining cardiovascular diseases, and the study by Zheng et al. demonstrated that an imbalance of testosterone/estradiol promotes male cardiovascular disease development [3]. Age increase is also a risk factor for the development of three-vessel coronary lesion and has a greater effect on the three-vessel coronary lesion in males than in females, as revealed by our study. Advanced age is a major risk factor for symptomatic and silent atherosclerosis disease and the aging process causes structural and functional alterations in the vascular wall, including intimal thickness, elevated arterial stiffness, and endothelial dysfunction [31]. Aging may make vascular walls more susceptible to hypercholesterolemia, plaque growth, and intra-plaque bleeding. With aging, the coronary plaque burden, necrotic core, and calcium content all increase significantly [32].

Our study may have some limitations including one center study focusing only on the Chinese population. Non-randomization is also one limitation of this study. In the future, a prospective randomized controlled study is needed to explore the possible risk factors for three-vessel coronary lesion.

In conclusion, the three-vessel coronary lesion is more prevalent in subjects with greater volume of epicardial fat and in the male gender. Epicardial fat, male gender, age increase, low HDL, high LDL, and increased fasting blood glucose are all risk factors for the prevalence of three-vessel coronary lesion.

## Data Availability

The data and materials are available from the corresponding author on reasonable request.

## References

[1] Jain S, Sarkar NC, Sarkar P, Modi N, Tilkar M (2015). Evaluation of coronary artery status by coronary angiography after first survival of acute myocardial infarction. J Clin Diagn Res.

[2] Papazoglou AS, Farmakis IT, Zafeiropoulos S, Moysidis DV, Karagiannidis E, Stalikas N (2022). Angiographic severity in acute coronary syndrome patients with and without standard modifiable risk factors. Front Cardiovasc Med.

[3] Zheng HY, Li Y, Dai W, Wei CD, Sun KS, Tong YQ (2012). Imbalance of testosterone/estradiol promotes male CHD development. Biomed Mater Eng.

[4] Shambu SK, Desai N, Sundaresh N, Babu MS, Madhu B, Gona OJ (2020). Study of correlation between epicardial fat thickness and severity of coronary artery disease. Indian Heart J.

[5] Ferreira J, Martins R, Monteiro S, Teixeira R, Goncalves L (2022). Alternative sites of echocardiographic epicardial fat assessment and coronary artery disease. J Ultrasound.

[6] Wang Q, Chi J, Wang C, Yang Y, Tian R, Chen X (2022). Epicardial adipose tissue in patients with coronary artery disease: a meta-analysis. J Cardiovasc Dev Dis.

[7] Iacobellis G (2022). Epicardial adipose tissue in contemporary cardiology. Nat Rev Cardiol.

[8] Karampetsou N, Alexopoulos L, Minia A, Pliaka V, Tsolakos N, Kontzoglou K (2022). Epicardial adipose tissue as an independent cardiometabolic risk factor for coronary artery disease. Cureus.

[9] Hirata Y, Tabata M, Kurobe H, Motoki T, Akaike M, Nishio C (2011). Coronary atherosclerosis is associated with macrophage polarization in epicardial adipose tissue. J Am Coll Cardiol.

[10] Lee KC, Yong HS, Lee J, Kang EY, Na JO (2019). Is the epicardial adipose tissue area on non-ECG gated low-dose chest CT useful for predicting coronary atherosclerosis in an asymptomatic population considered for lung cancer screening?. Eur Radiol.

[11] Lin A, Wong ND, Razipour A, McElhinney PA, Commandeur F, Cadet SJ (2021). Metabolic syndrome, fatty liver, and artificial intelligence-based epicardial adipose tissue measures predict long-term risk of cardiac events: a prospective study. Cardiovasc Diabetol.

[12] Koplev S, Seldin M, Sukhavasi K, Ermel R, Pang S, Zeng L (2022). A mechanistic framework for cardiometabolic and coronary artery diseases. Nat Cardiovasc Res.

[13] Gavalda-Navarro A, Villarroya J, Cereijo R, Giralt M, Villarroya F (2022). The endocrine role of brown adipose tissue: An update on actors and actions. Rev Endocr Metab Disord.

[14] Martins FF, Souza-Mello V, Aguila MB, Mandarim-de-Lacerda CA (2022). Brown adipose tissue as an endocrine organ: updates on the emerging role of batokines. Horm Mol Biol Clin Investig.

[15] Greif M, Becker A, von Ziegler F, Lebherz C, Lehrke M, Broedl UC (2009). Pericardial adipose tissue determined by dual source CT is a risk factor for coronary atherosclerosis. Arterioscler Thromb Vasc Biol.

[16] Jeong JW, Jeong MH, Yun KH, Oh SK, Park EM, Kim YK (2007). Echocardiographic epicardial fat thickness and coronary artery disease. Circ J.

[17] Kim TH, Yu SH, Choi SH, Yoon JW, Kang SM, Chun EJ (2011). Pericardial fat amount is an independent risk factor of coronary artery stenosis assessed by multidetector-row computed tomography: the Korean atherosclerosis study 2. Obesity.

[18] Chaowalit N, Somers VK, Pellikka PA, Rihal CS, Lopez-Jimenez F (2006). Subepicardial adipose tissue and the presence and severity of coronary artery disease. Atherosclerosis.

[19] Oba S, Suzuki E, Nishimatsu H, Kumano S, Hosoda C, Homma Y (2012). Renoprotective effect of erythropoietin in ischemia/reperfusion injury: possible roles of the Akt/endothelial nitric oxide synthase-dependent pathway. Int j urol.

[20] Mazurek T, Zhang L, Zalewski A, Mannion JD, Diehl JT, Arafat H (2003). Human epicardial adipose tissue is a source of inflammatory mediators. Circulation.

[21] Turkmen K, Ozer H, Kusztal M (2022). The relationship of epicardial adipose tissue and cardiovascular disease in chronic kidney disease and hemodialysis patients. J Clin Med.

[22] Ouchi N, Kihara S, Arita Y, Okamoto Y, Maeda K, Kuriyama H (2000). Adiponectin, an adipocyte-derived plasma protein, inhibits endothelial NF-kappaB signaling through a cAMP-dependent pathway. Circulation.

[23] Zhang Z, Du J, Xu Q, Xing C, Li Y, Zhou S (2022). Adiponectin suppresses metastasis of nasopharyngeal carcinoma through blocking the activation of NF-kappaB and stat3 signaling. Int J Mol Sci.

[24] Broedl UC, Lebherz C, Lehrke M, Stark R, Greif M, Becker A (2009). Low adiponectin levels are an independent predictor of mixed and non-calcified coronary atherosclerotic plaques. PLoS ONE.

[25] Tabas I, Williams KJ, Boren J (2007). Subendothelial lipoprotein retention as the initiating process in atherosclerosis: Update and therapeutic implications. Circulation.

[26] Perrotta I (2022). Atherosclerosis: from molecular biology to therapeutic perspective. Int J Mol Sci.

[27] Voros S, Joshi P, Qian Z, Rinehart S, Vazquez-Figueroa JG, Anderson H (2013). Apoprotein b, small-dense LDL and impaired HDL remodeling is associated with larger plaque burden and more noncalcified plaque as assessed by coronary CT angiography and intravascular ultrasound with radiofrequency backscatter: results from the ATLANTA I study. J Am Heart Assoc.

[28] Grant PJ (2007). Diabetes mellitus as a prothrombotic condition. J Intern Med.

[29] Wehr E, Pilz S, Boehm BO, Marz W, Grammer T, Obermayer-Pietsch B (2011). Low free testosterone is associated with heart failure mortality in older men referred for coronary angiography. Eur J Heart Fail.

[30] Reichelt ME, Mellor KM, Bell JR, Chandramouli C, Headrick JP, Delbridge LM (2013). Sex, sex steroids, and diabetic cardiomyopathy: making the case for experimental focus. Am j physiol Heart circ physiol.

[31] Lakatta EG, Levy D (2003). Arterial and cardiac aging: major shareholders in cardiovascular disease enterprises: part I: aging arteries: a "set up" for vascular disease. Circulation.

[32] Ruiz-Garcia J, Lerman A, Weisz G, Maehara A, Mintz GS, Fahy M (2012). Age- and gender-related changes in plaque composition in patients with acute coronary syndrome: the PROSPECT study. EuroIntervention.

